# Co-detection of Panton-Valentine leukocidin encoding genes and cotrimoxazole resistance in *Staphylococcus aureus* in Gabon: implications for HIV-patients’ care

**DOI:** 10.3389/fmicb.2015.00060

**Published:** 2015-02-05

**Authors:** Christian Kraef, Abraham S. Alabi, Georg Peters, Karsten Becker, Peter G. Kremsner, Elie G. Rossatanga, Alexander Mellmann, Martin P. Grobusch, Philipp Zanger, Frieder Schaumburg

**Affiliations:** ^1^Institute of Medical Microbiology, University Hospital MünsterMünster, Germany; ^2^Centre de Recherches Médicales de Lambaréné, Albert Schweitzer HospitalLambaréné, Gabon; ^3^Institut für Tropenmedizin, Eberhard Karls Universität, Deutsches Zentrum für InfektionsforschungTübingen, Germany; ^4^Centre de Traitement AmbulatoireLambaréné, Gabon; ^5^Institute of Hygiene, University Hospital MünsterMünster, Germany; ^6^Division of Internal Medicine, Center of Tropical Medicine and Travel Medicine, Department of Infectious Diseases, Academic Medical Center, University of AmsterdamAmsterdam, Netherlands; ^7^Institute of Public Health, University Hospital HeidelbergHeidelberg, Germany

**Keywords:** *Staphylococcus aureus*, Panton-Valentine leukocidin, cotrimoxazole resistance, Africa, HIV

## Abstract

Patients infected with the human immunodeficiency virus (HIV) are frequently exposed to antimicrobial agents. This might have an impact on the resistance profile, genetic background and virulence factors of colonizing *Staphylococcus aureus*. Sub-Saharan Africa is considered to be endemic for Panton-Valentine leukocidin (PVL) positive *S. aureus* which can be associated with skin and soft tissue infections (SSTI). We compared *S. aureus* from nasal and pharyngeal swabs from HIV patients (*n* = 141) and healthy controls (*n* = 206) in Gabon in 2013, and analyzed determinants of colonization with PVL positive isolates in a cross-sectional study. *S. aureus* isolates were screened for the presence of selected virulence factors (incl. PVL) and were subjected to antimicrobial susceptibility testing and genotyping. In HIV patients, *S. aureus* was more frequently detected (36.9 vs. 31.6%) and the isolates were more frequently PVL positive than in healthy controls (42.1 vs. 23.2%). The presence of PVL was associated with cotrimoxazole resistance (OR = 25.1, *p* < 0.001) and the use of cotrimoxazole was a risk factor for colonization with PVL positive isolates (OR = 2.5, *p* = 0.06). PVL positive isolates were associated with the multilocus sequence types ST15 (OR = 5.6, *p* < 0.001) and ST152 (OR = 62.1, *p* < 0.001). Participants colonized with PVL positive isolates reported more frequently SSTI in the past compared to carriers of PVL negative isolates (OR = 2.7, *p* = 0.01). In conclusion, the novelty of our study is that cotrimoxazole might increase the risk of SSTI in regions where cotrimoxazole resistance is high and associated with PVL. This finding needs to be confirmed in prospective studies.

## INTRODUCTION

*Staphylococcus aureus* is a bacterial pathogen causing a broad range of infections such as skin and soft tissue infections (SSTI), osteomyelitis, pneumonia, and endocarditis (Wertheim et al., 2005). Nasal colonization often precedes invasive infection which is frequently caused by the homolog isolate of the anterior nares (von Eiff et al., 2001). Therefore, nasal colonization rates and molecular characterization of colonizing isolates provide important insights into the risk of *S. aureus* infection in selected human populations. A recent study showed that impairment of the immune system by the human immunodeficiency virus (HIV) increases the risk for *S. aureus* colonization (Olalekan et al., 2012).

In the present study we compare *S. aureus* from HIV patients and healthy controls as *S. aureus* belongs to the most relevant pathogens of both superficial and invasive infections in Africa (Reddy et al., 2010; Alabi et al., 2013).

*Staphylococcus aureus* possess an armory of different virulence factors (Otto, 2014b). The Panton-Valentine leukocidin (PVL) is one of the most prevalent virulence determinant in *S. aureus* in sub-Saharan Africa (Schaumburg et al., 2014b). PVL is a bi-component protein toxin that binds to the complement receptor C5a of neutrophils (Spaan et al., 2013). This leukocidin shows strong cytotoxicity on granulocytes and can be associated with severe SSTI (Shallcross et al., 2013). However, its role in pathogenesis in humans remains controversial (Otto, 2014b).

Sub-Saharan Africa suffers from the highest burden of HIV infections in the world; 68% of all infected persons reside in this region, which only accounts for 12% of the global population (World Health Organization, 2011). Cotrimoxazole (trimethoprim-sulfamethoxazole) prophylaxis has been shown to lower mortality in HIV infected patients through reduced rates of *Pneumocystis jirovecii* pneumonia, malaria, and bacterial infections (Suthar et al., 2012; Bwakura-Dangarembizi et al., 2014). However, cotrimoxazole is also a broad spectrum antibacterial drug which might impact on *S. aureus* colonization of HIV patients particularly in long-term use. Cotrimoxazole inhibits the folate biosynthesis by blocking the dihydropteroate synthase (DHPS, sulfamethoxazole) and the dihydrofolate reductase (DHFR, trimethoprim; Lyon and Skurray, 1987). Cotrimoxazole resistance is caused by mutations of the genes encoding the DHPS and/or DHFR or the acquisition of mutated genes through horizontal gene transfer (Lyon and Skurray, 1987). A recent study showed that the DHFR gene *dfrG* is the major cause of trimethoprim resistance in sub-Saharan Africa (Nurjadi et al., 2014).

In the present study, we (i) compared *S. aureus* isolates from HIV positive patients with those from healthy controls and (ii) analyzed determinants of colonization with PVL positive isolates. These study objectives are novel as they address for the first time determinants of colonization with PVL positive *S. aureus* in Africa with a special focus on HIV patients which are frequently exposed to antimicrobial agents.

## MATERIALS AND METHODS

### ETHICAL CLEARANCE

Ethical approval was obtained from the institutional review board of the “Centre de Recherches Médicales de Lambaréné” in Gabon (CEI 004/2013). Written informed consent was signed by all participants before enrollment.

### STUDY DESIGN

The primary objective of the study was to compare resistance rates and virulence factors of *S. aureus* from HIV patients with those from healthy controls. The secondary objective was to identify risk factors for the colonization with PVL positive isolates.

A recent study from Nigeria showed mean resistance rates to antimicrobial agents (i.e., cotrimoxazole, chloramphenicol, and ciprofloxacin) in *S. aureus* from HIV positive patients and healthy controls of 51.7 and 23.7%, respectively (Olalekan et al., 2012). A sample size calculation assuming these mean resistance rates, a power of 0.8 and an alpha of 0.05 gave a total of 53 isolates per group. Assuming an average colonization rate of 37% in HIV positive patients and 25% in healthy people yielded a sample size of 143 HIV patients and 212 healthy controls (Shet et al., 2009; Olalekan et al., 2012; Kotpal et al., 2014).

Participants were recruited from the Lambaréné region in Gabon between March and September 2013 if they met the inclusion criteria of (i) providing written informed consent and (ii) age ≥18 years. Subjects with culture confirmed *S. aureus* infections at enrollment were excluded from the final analysis.

Human immunodeficiency virus positive patients attending one of the three out-patient clinics in Lambaréné (Hôpital Albert Schweitzer, Hôpital Régional George Rawiri, Centre de Traitement Ambulatoire) were recruited. Healthy controls were recruited in Lambaréné and surrounding villages.

An HIV test was offered to each participant to verify the HIV status. Demographic data (sex, age), medical history (hospitalization, use of antibiotics in the past 6 months, history of SSTIs), were documented in a standardized questionnaire. The patient’s information was derived from records of the personal health care files and interviews.

### HIV TEST AND CD4^+^ CELL COUNT

Human immunodeficiency virus status was assessed using rapid tests (Determine HIV1/2, Abbott Laboratories, Tokyo, Japan or Vikia, bioMérieux, Marcy l’Etoile, France). T-helper cells [cluster of differentiation four positive cells (CD4^+^ cells)] were measured in venous EDTA blood using FACSCount (BD Biosciences, San Jose, CA, USA).

### BACTERIAL ISOLATES

At enrollment, one nasal swab was taken by swabbing gently the mucosa of both anterior nares (including the nasal septum) for few seconds with rotating movements (Schaumburg et al., 2014a). One pharyngeal swab was taken by swabbing the posterior pharyngeal wall with horizontal movements (Schaumburg et al., 2014a). Cotton tips were stored in Amies medium (Transwabs, Medical Wire, Corsham, UK) and cultured within 3 h for 24–48 h on Columbia blood agar supplemented with aztreonam disks (13 μg; Oxoid, Hants, UK; Schaumburg et al., 2014a).

Colonies were tested for positive catalase and agglutination test (Pastorex, Staph-Plus; Bio-Rad Laboratories, Marnes-la-Coquette, France). Species identification and antimicrobial susceptibility test (EUCAST clinical breakpoints) were done using Vitek 2 automated systems (bioMérieux). Species was confirmed by the PCR detection of the thermostable nuclease *nuc* (Brakstad et al., 1992). Methicillin resistance was confirmed by the PCR detection of the penicillin binding protein 2a (PBP2a) encoding gene *mecA* (Becker et al., 2006). All MRSA were screened for the arginine catabolic mobile element (ACME; Diep et al., 2006).

Genes encoding the exfoliative toxins, pyrogenic toxin superantigens (i.e., enterotoxins, toxic shock syndrome toxin) and the Panton-Valentine leukocidin (PVL) were detected by PCR (Lina et al., 1999; Becker et al., 2003).

If a participant was colonized with an identical isolate [based on *spa* typing (Mellmann et al., 2006)] in the nose and throat, only one isolate was included in the final analysis of resistance rates and virulence factors in order to report non-duplicate isolates only.

### GENOTYPING

All isolates were *S. aureus* protein A (*spa*) typed based on the sequence of the hypervariable region of protein A (Mellmann et al., 2006). One isolate of each *spa* type was subjected to multilocus sequence typing (MLST; Enright et al., 2000) and each MLST sequence type (ST) was assigned to MLST clonal complexes (CC) using eBURST^1^.

### STATISTICS

Colonization was defined as detection of *S. aureus* in the anterior nares and/or pharynx. The χ^2^- and Fisher exact tests and logistic regression analyses were used to assess the association of HIV status with demographic characteristics and molecular features of *S. aureus* (e. g. antimicrobial resistance, virulence factors), to estimate odds ratio (OR), the 95% confidence intervals (95% CI) and to adjust for potential confounders. The significance level was set at 0.05. Mean values are shown ± SD. Statistical analysis was performed with “R” and the package “epicalc^2^.”

To test if statistical associations of the present study could be reproduced, we used a database including 469 non-duplicate isolates (based on *spa* typing) from healthy, HIV negative, asymptomatic volunteers collected during earlier studies in the Lambaréné region (Schaumburg et al., 2013, 2014a).

## RESULTS

### STUDY POPULATION

In total, 348 participants were recruited; one HIV positive patient was excluded due to a culture confirmed *S. aureus* wound infection at inclusion. Therefore, 141 HIV positive participants and 206 healthy controls were included in the final analysis.

All participants in the HIV group were confirmed to be HIV positive. The median (range) CD4^+^ cell count was 166 (2-1374) cells/μl. In total, 49 HIV patients (34.8%) were on antiretroviral therapy at the time of inclusion.

In the control group, 68% (*n* = 140) were HIV negative, 26.9% (*n* = 56) had a negative test result within the last 3 months but refused a confirmatory test, and 4.9% (*n* = 10) did not report any test results but refused to be tested for HIV.

### COMPARISON OF *S. aureus* COLONIZATION BETWEEN HIV PATIENTS AND HEALTHY CONTROLS

On average, participants in both comparison groups were of the same age (**Table 1**). However, HIV positive subjects were more often female and reported more health care contacts within the past 6 months compared to healthy controls (**Table 1**). HIV patients significantly used more antimicrobial agents than healthy controls in the past 6 months; such as cotrimoxazole (including cotrimoxazole prophylaxis, 34.0 vs. 1.5%, OR = 33.3, 95% CI: 11.1–100.0, *p* < 0.001), amoxicillin/clavulanic acid (22.7 vs. 8.3%, OR = 3.2, 95% CI: 1.7–6.7, *p* < 0.001) and ciprofloxacin (9.9 vs. 1.5%, OR = 7.7, 95% CI: 2.0–50.0, *p* = 0.001). No association of *S. aureus* colonization and CD4^+^ cell count was detected (data not shown).

**Table 1:** Baseline characteristics of the study population.

	HIV positive participants (*n* = 141)^a^	Healthy controls (*n* = 206)^a^	OR (95% CI)	*p*
Age, mean ± SD	38.7 ± 11.1	37.5 ± 15.5	1 (1.0–1.0)	0.4
Sex, female	102 (72.3%)	120 (58.3%)	1.8 (1.2–2.9)	0.01
Hospitalization in the past 6 months	95 (67.4%)	46 (22.3%)	0.1 (0.1–0.2)	<0.001
Antibiotics in the past 6 months	107 (75.9%)	61 (29.6%)	0.1 (0.1–0.2)	<0.001
Pyodermatitis	29 (20.6%)	13 (6.3%)	0.3 (0.1–0.6)	<0.001
*S. aureus* colonization	52 (36.9%)	65 (31.6%)	0.8 (0.5–1.2)	0.3
Nasal *S. aureus* colonization	36 (25.5%)	40 (19.4%)	0.7 (0.4–1.2)	0.2
Pharyngeal *S. aureus* colonization	29 (20.6%)	37 (18.0%)	0.8 (0.5–1.4)	0.5

*Data are n (%) of subjects unless otherwise indicated, OR (95%CI) and *p*-value derived from logistic regression analysis.*

*^a^A pharyngeal swab was refused by three HIV positive participants and one control.*

*Staphylococcus aureus* colonization was associated with HIV positive participants but did not reach statistical significance (**Table 1**). This association remained stable when adjusting carriage for history of pyodermatitis, hospitalization and use of antibiotics in the past 6 months (adjusted OR = 0.8, 95% CI: 0.5–1.2, *p* = 0.3).

In total, 125 non-duplicate isolates from HIV patients (*n* = 56) and healthy controls (*n* = 69) were included in the final analysis. Compared to healthy controls, resistance rates of cotrimoxazole were slightly higher in isolates from HIV patients, but did not reach statistical significance (**Table 2**). The distribution of resistance rates of other tested antimicrobial agents was similar in both groups (**Table 2**). No resistance was detected against clindamycin, linezolid, fosfomycin, fusidic acid, and glycopeptides. For tested virulence factors, PVL was more frequently detected in isolates from HIV patients compared to healthy controls (42.9 vs. 23.2%, **Table 2**). Genes encoding other virulence factors such as the enterotoxins *sea*, *seg*, and *sec*, the exfoliative toxin a (*eta*) and the toxic shock syndrome toxin (*tst*) were equally distributed among both groups.

**Table 2:** Characteristics of *Staphylococcus aureus* in HIV patients and healthy controls, Gabon.

		*S. aureus,* n (%)		OR (95%CI)	*P*
		HIV positive participants (*n* = 56)	Healthy controls (*n* = 69)		
Antimicrobial resistance	Penicillin	52 (92.9%)	66 (95.7%)	0.6 (0.1–3.7)	0.5
	Oxacillin	3 (5.4%)	2 (2.9%)	1.9 (0.2–23.4)	0.7
	Erythromycin	4 (7.1%)	10 (14.5%)	0.5 (0.1–1.7)	0.3
	Gentamicin	1 (1.8%)	1 (1.5%)	1.2 (0.0–98.4)	1
	Tetracyclin	26 (46.4%)	30 (43.5%)	1.1 (0.5–2.4)	0.7
	Cotrimoxazole	32 (57.1%)	33 (47.8%)	1.4 (0.7–3.3)	0.3
Virulence factors	*luk*S-PV/*luk*F -PV (PVL)	24 (42.9%)	16 (23.2%)	2.5 (1.1–5.9)	0.02
	*sea*	18 (32.1%)	17 (24.6%)	1.5 (0.6–3.5)	0.4
	*seg*	15 (26.8%)	22 (31.9%)	0.8 (0.3–1.8)	0.5
	*sec*	6 (10.7%)	6 (8.7%)	1.3 (0.3–5)	0.7
	*eta*	2 (3.6%)	1 (1.5%)	2.5 (0.1–100)	0.6
	*tst*	3 (5.4%)	3 (4.4%)	1.3 (10)	1

The MRSA isolates belonged to *spa* types t121 (*n* = 1, ST8, PVL positive, ACME positive), t451 (*n* = 1, ST8, PVL negative, ACME positive), t1476 (*n* = 1, ST6, PVL negative, ACME negative) and t12858 (*n* = 2, PVL negative, ACME positive). **Table 3** shows the distribution of genotypes in all *S. aureus* isolates from HIV patients and healthy controls.

**Table 3:** Genotypes of *Staphylococcus aureus* isolates from HIV patients and healthy controls, in Gabon in 2013.

CC	ST	Isolates from HIV-positive participants (*n* = 56)		Isolates from healthy controls (*n* = 69)		OR (95% CI)^a^	*P*^a^
		*S. aureus spa* types (*n*)	Total	*S. aureus spa* types (*n*)	Total		
CC5	ST1	t127 (1)	64.9%	t127 (3), **t4448** (1)	72.1%	1.32 (0.58–3.01)	0.5
	ST5	t5987 (1)		t319 (3), t1400 (2), t5987 (4)			
	ST6	t701 (1), t1476 (3)		t304 (1), t701 (1), t1476 (4)			
	ST8	**t121** (1), t064 (1), t12858 (2)		t451 (1), t1476 (2)			
	ST9	t1045 (1)		t1045 (1)			
	ST15	**t084** (15), **t094** (1), **t491** (2), **t803** (1)		**t084** (12), **t085** (1), **t279** (2), t12523 (1)			
	ST72	t148 (3)		t148 (3), t2464 (1), t11108 (1)			
	ST188	t189 (1)		t189 (1)			
	ST573	t1579 (1)		–			
	ST2708	t311 (1)		t311 (3)			
	ST2736	–		t254 (1)			
	ST2765	t11644 (1)		–			
CC45	ST45	t939 (2)	7.0%	t12857 (1)	4.4%	0.6 (0.08-3.74)	0.7
	ST508	t015 (1), t073 (1)		t12525 (1)			
	ST2498	–		t827 (1)			
CC101	ST101	t056 (1)	1.8%	t056 (4), t3165 (1), t4679 (2)	10.3%	6.32 (0.76-289.54)	0.07
CC152	ST152	**t355** (10)	17.5%	**t355** (8)	11.8%	0.62 (0.2-1.9)	0.3
Other	ST7	t091 (1)	8.8%	–	1.5%	0.31 (0.03-2.01)	0.2
	ST88	t12856 (1)		t2723 (1)			
	ST121	t645 (1)		–			
	ST2349	t026 (1)		–			
	ST1223	–		NT (1)			

*PVL, positive *spa* types in bold. ^a^Comparison of the proportion of clonal complexes (CC).*

### DETERMINANTS OF COLONIZATION WITH PVL POSITIVE *S. aureus*

We now addressed potential variables for the colonization with PVL positive *S. aureus* and hypothesized that the use of antimicrobial agents might select for PVL positive isolates. For this purpose, we merged isolates from HIV patients and healthy controls. PVL positive isolates were significantly associated with cotrimoxazole resistance (**Table 4**). Use of cotrimoxazole was significantly associated with cotrimoxazole resistance (OR = 3.3, 95% CI: 1.0–12.4, *p* = 0.03). This was in line with an association of cotrimoxazole use and the detection of PVL positive isolates but did not reach statistical significance (**Table 4**). PVL positive isolates belonged more likely to ST15 and ST152 (**Table 4**). Cotrimoxazole resistance was strongly associated with ST15 (OR = 18.4, 95% CI: 5.1–99.0, *p* < 0.001) and ST152 (OR = 5.7, 95% CI: 1.5–32.1, *p* = 0.004). The colonization with either of these genotypes were more common in HIV positive patients compared to healthy controls (OR = 2.0, 95% CI: 0.9–4.4, *p* = 0.06) and in participants reporting use of cotrimoxazole (OR = 2.3, 95% CI: 0.8, 7.2, *p* = 0.08).

**Table 4:** Determinants of colonization with PVL positive *S. aureus.*

Characteristics	PVL positive *S. aureus* (*n* = 40)	PVL negative *S. aureus* (*n* = 85)	Crude OR (95%CI)	*P*	Adjusted OR^c^ (95%CI)	*P*^c^
Use of antimicrobials	26 (65.0%)	36 (42.4%)	2.5 (1.1–6.0)	0.02	2.0 (0.8–4.6)	0.13
Cotrimoxazole use^a^	10 (25.0%)	10 (11.8%)	2.5 (0.8–7.4)	0.06		
Abscess^a^	11 (27.5%)	13 (15.3%)	2.1 (0.8–5.7)	0.1		
Any SSTI^a,b^	20 (50.0%)	23 (27.1%)	2.7 (1.1–6.3)	0.01	2.6 (1.2–5.7)	0.02
Cotrimoxazole resistance	37 (92.5%)	28 (32.9%)	25.1 (6.9–134.4)	<0.001	26.0 (7.2–93.8)	<0.001
*S. aureus* ST15	21 (52.5%)	14 (16.5%)	5.6 (2.2–14.2)	<0.001	5.4 (2.3–12.7)	<0.001
*S. aureus* ST152	17 (42.5%)	1 (1.2%)	62.1 (8.5–2584.6)	<0.001	65.6 (8.1–532.7)	<0.001

*Data are n (%) of subjects. SSTI, skin and soft tissue infection. Eight carriers were colonized with two different isolates resulting in 125 isolates from 117 colonized individuals. ^a^in the past 6 months. ^b^any SSTI: wound infection, carbuncle/furuncle, abscess, pyodermatitis, ^c^adjusted for HIV infection.*

Of note, participants colonized with PVL positive *S. aureus* reported more frequently any type of SSTIs (pyodermatitis, abscess, furuncle/carbuncle, wound infection) in the past 6 months than those colonized with other isolates (**Table 4**).

As HIV infection might be a confounder in our dataset we performed a multivariate analysis and adjusted those association for HIV status which were statistically significant in the univariate analysis. HIV infection was a weak confounder of the association between the use of antimicrobials and the detection of PVL positive isolates (**Table 4**). The associations between PVL and SSTI, cotrimoxazole resistance, ST15 and ST152 remained stable after adjusting for HIV status.

As cotrimoxazole resistant isolates were associated with PVL, we analyzed the association of different exposure variables with the combined outcome “PVL positivity and cotrimoxazole resistance.” This combined outcome was also significantly associated with the exposure “use of antimicrobial agents” (OR = 2.4, 95% CI: 1.1–5.4, *p* = 0.03) and “SSTI” (OR = 2.4, 95% CI: 1.1–5.3, *p* = 0.03) in the past 6 months.

### ASSOCIATION OF PVL POSITIVITY AND COTRIMOXAZOLE RESISTANCE IN A RETROSPECTIVE STRAIN COLLECTION

We now tested if our findings could be reproduced analyzing an already existing strain collection of 469 non-duplicate isolates from healthy, HIV negative, asymptomatic volunteers from earlier studies in the Lambaréné region (Schaumburg et al., 2013, 2014a). Here, we also found strong evidence for an association of cotrimoxazole resistance with the presence of PVL in colonizing *S. aureus* (OR = 5.9, 95% CI: 3.7–9.1, *p* < 0.001) and with the genotypes ST152 or ST15 (OR = 14.3, 95%CI: 9.1–25.0, *p* < 0.001, Table S1).

## DISCUSSION

Our study found an association of PVL positive isolates with the use of cotrimoxazole and cotrimoxazole resistance. These findings suggest a selection of cotrimoxazole resistant PVL positive *S. aureus* clones through the use of cotrimoxazole. Use of cotrimoxazole might therefore put individuals at higher risk for SSTI in regions where cotrimoxazole resistance is high and associated with PVL. This might be particularly true for HIV patients receiving long term cotrimoxazole prophylaxis.

The higher proportion of females in the HIV group has been reported by others as well and might either reflect a higher risk for HIV infection in females (Olalekan et al., 2012) or gender differences in the overall willingness to participate in research that requires disclosure of HIV status. We detected higher *S. aureus* colonization rates in HIV patients compared to healthy controls, which is in line with a recent report from Nigeria (Olalekan et al., 2012) and a 3–7 fold higher prevalence of HIV infection in adult women compared to men (Karim et al., 2010).

It is well established that the use of antimicrobial agents selects for resistant isolates (Monroe and Polk, 2000; van de Sande-Bruinsma et al., 2008). Therefore, it is not surprising that cotrimoxazole resistance is higher in African HIV patients who are more frequently exposed to cotrimoxazole, as shown in our study. However, cotrimoxazole is also amongst the most frequently used antimicrobial compounds in the general population which explains the high cotrimoxazole resistance in Gabon and other regions of Africa (up to 55% in colonizing isolates; Schaumburg et al., 2014b).

We showed that the use of cotrimoxazole is a risk factor for colonization with PVL positive and cotrimoxazole resistant ST15 and ST152 isolates. These two lineages are the major PVL positive clones in sub-Saharan Africa (Schaumburg et al., 2014b). Co-occurrence of cotrimoxazole resistance with PVL or with the MLST genotypes ST15 and ST152 was confirmed by reanalyzing published data from healthy asymptomatic carriers in Gabon (Schaumburg et al., 2013, 2014a). It is possible that this finding can be extrapolated to other African regions where PVL is associated with cotrimoxazole resistance as well (Breurec et al., 2011).

Altogether, our analysis suggests the following model: cotrimoxazole resistance co-occurs with PVL. The use of cotrimoxazole selects for cotrimoxazole resistant isolates which are frequently PVL positive. The possession of PVL and most likely other virulence factors might pose a risk for SSTI.

However, it remains unclear why cotrimoxazole resistance is co-detected with PVL. One might speculate that genes encoding PVL and cotrimoxazole resistance are carried by the same bacteriophage which has transferred both determinants to the bacterial host genome particularly to *S. aureus* belonging to ST15 and ST152. One might then expect that both genes are located in close proximity to each other. However, to the best of our knowledge, no PVL bacteriophage has been described which also carries antimicrobial resistance genes. Only one novel PVL bacteriophage has been reported which also carried the enterotoxin a gene (*sea*; Prabhakara et al., 2013). Whole genome sequencing should be performed in future (i) to map the genes of PVL and cotrimoxazole resistance in the bacterial genome and (ii) to assess if both are carried by the same bacteriophage.

Despite these findings we would like to point out that cotrimoxazole prophylaxis should not be discouraged in HIV patients as the benefit has been clearly shown (Suthar et al., 2012; Bwakura-Dangarembizi et al., 2014), and as clinical implications of PVL are still controversial (Otto, 2014b). The implication of our study on HIV patients’ care is to sensitize physicians for the counselling on and the early detection of SSTI in patients receiving cotrimoxazole prophylaxis. A higher risk of SSTI in these patients might be possible as our analysis revealed an association of PVL positive isolates with a history of SSTI in the past 6 months that was independent of the HIV status. In addition, a strong association of PVL with SSTIs was also recently shown by a large meta-analysis (Shallcross et al., 2013).

However, PVL is not the only virulence factor which can be associated with SSTIs. Strong tissue toxicity has also been shown for the α-toxin or the phenol-soluble modulins (Becker et al., 2014; Otto, 2014a).

Our study is limited by our small sample size and an unknown HIV status of 10 (4.9%) participants. We believe that the risk of miss-including HIV positive patients in the control group is low as the HIV prevalence was low in Gabon in 2009 (5.2%, declining trend) compared to South and East Africa (World Health Organization, 2011). In addition these participants were not followed by any of the HIV clinics in Lambaréné. Second, the setting of the recruitment was not the same for HIV positive patients (clinics) and controls (community setting). As only healthy controls should be included, we were not able to recruit them in the hospital setting. Third, the cross-sectional study design cannot detect the causality of high prevalence of PVL positive isolates. We only are able to identify potential factors causing a selection of PVL positive isolates which might be addressed in future prospective studies.

In conclusion, cotrimoxazole use was associated with PVL positive and cotrimoxazole-resistant *S. aureus*. Therefore, cotrimoxazole prophylaxis may put HIV patients at risk of SSTIs in regions where cotrimoxazole resistance is high and associated with PVL.

## Conflict of Interest Statement

The authors declare that the research was conducted in the absence of any commercial or financial relationships that could be construed as a potential conflict of interest.

## Footnotes

1 http://saureus.mlst.net; hosted at the Imperial College, London

2 http://cran.r-project.org

## SUPPLEMENTARY MATERIAL

The Supplementary Material for this article can be found online at:
